# Dysmenorrhœa

**Published:** 1833

**Authors:** 


Art. IV. *Dysmenorrhœa.*—We are indebted to the Boston Med. and Surg. Journal of August last, for a paper on this painful and obstinate affection, by Mr. Wilson, of Glasgow, Scotland. We presume that few of our readers have not been baffled in their attempts at curing this affection, or even relieving the sufferings of their patients. For ourselves, we are compelled to say, that we have neither definite ideas of its pathology, nor a knowledge of any remedy on which reliance can be placed. In regard to the former, Mr. Wilson holds this language:

"1st. There may be such a degree of structural derangement of the uterus, as either to unfit it altogether for menstruating, or to permit the function to be performed, but not without pain and difficulty.

2dly. An inflated or irritable state of the uterus, its nerves or its blood vessels, may give rise to painful menstruation.

3dly. There may, in certain individuals, be painful menstruation, in consequence of either local or general debility. In such, the circulation is so weak that the extremities of the uterine arteries are not easily forced open, or a degree of unnatural constriction or spasm of these extremities exists, which resists, and that painfully, the ordinary or enfeebled impulse of the circulation.

Or, 4thly. There may be, as Dr. Macintosh supposes, complete closure of the os uteri preventing the escape of the menstruous fluid, or the orifice may be so very small as to render its escape difficult and painful."

Of the cases depending on the first of these conditions, our author speaks as follows:

"Painful menstruation arising from this cause, is, I presume, most frequently met with in those who are somewhat advanced in life. Childbearing subjects the uterus to many accidents and much rudeness, which lay the foundation of structural derangements at some subsequent period. In such cases, however desirable it might be to ascertain the exact state of the uterus, this will in general be beyond our reach; and even were such a diagnosis attainable, the present state of our knowledge and experience furnishes almost nothing for the removal of such uterine disease."

On the second course, he has the subjoined comment:

"The second cause mentioned of painful menstruation was an inflamed or irritable state of the uterus, its nerves or its blood vessels. Dr. Blondell says dysmenorrhœa is not an inflammatory affection. I believe it is not so in general; yet there are many cases which bear strong marks of an inflammatory character, and it would appear that the uterine or menstrual vessels are at times so excited as to furnish a product similar in many respects to the buffy coat of inflamed blood.

It has been long known that many females who menstruate with pain, have discharged a membrane from the uterus similar in shape and structure to the membrane decidua, and that while the decidua is the effect of the excitement of conception, this menstruous membrane is the effect of an inflamed or similarly excited state of the uterine vessels. Dr. Denman was led, from the frequency with which he met this membrane, to ascribe difficult menstruation to it in general. I have met with this membraneous production in several instances, but in many other cases, although it has been sought for with the greatest possible care, not a vestige of it could be found. Where such a membrane exists, it is easy to perceive how menstruation should be difficult. There is a coating formed on the inner surface of the uterus, which will present a complete barrier to the easy effusion of the menstrual fluid, so that many painful efforts may be necessary by the obstructed vessels and the uterus generally, before this membrane is expelled.

In many cases when this membrane does not exist, and when, of course, there is no such obstacle offered to the escape of the menstruous fluid, menstruation will, I conceive, be found painful, from the mere circumstance of the uterine vessels discharging their contents in their inflamed state. It is very probable, that whenever there is inflammation, the blood vessels, as well as nerves, participate in it; and as the blood vessels are endowed with sensibility, we cannot avoid the conclusion that the chief source of pain, in many cases of local inflammation, arises from the arteries being under the necessity of acting in this their unnatural and diseased state."

The treatment of the cases referrible to this head, should of course be the antiphlogistic.

"Here bleeding, both local and general—warm hip-bath, with other autiphlogistic means, will be found most useful. In such cases much benefit might be expected from an alterative course of mercury, conjoined with opium."

On the third supposed cause, general or local weakness, without inflammation, Mr. W. makes several observations, of which the following are the conclusion.

I am convinced debility will be found far more frequently the cause of dysmenorrhœa than either structural derangement or an inflammatory state of the uterus; at all events, debility aud painful menstruation are very generally found together, so that even in cases where there has been good reason to suppose structural derangement or an inflamed state to have been the primary cause, debility has at length been superinduced by the severe and continued suffering from painful menstruation."

It is cases of this class that Chalybeate preparations and other metallic tonics, camphor and Dewees' tincture of Guaiacum are adopted. In regard to the last, our author's testimony accords precisely with our own experience. He has not been able to obtain by its use the beneficial effects which Dr. Dewees professes to have found from its exhibition, and pointedly condemns its employment in cases attended with inflammation. It may be remarked, however, that an inflammatory condition may be present during the menstrual period, but absent at all other times.

We have met with many cases of Dysmenorrhœa, in which during the paroxysms, the uterus seemed to labour under a real neuralgia. Would not cupping over the sacrum, or moxa to that part be attended with good effects? Might not injections into the uterus, of warm water, with or without narcotic extracts, afford relief in the paroxysm? We are not aware that these means have been fairly tried, and the last might be found inconvenient in practice.

"The fourth cause of dysmenorrhœa is partial or complete closure of the os uteri. Dr. M'Intosh of Edinburgh has lately given some important facts relative to this subject, and his opinion seems to be that the pain during menstruation is owing, in most cases, to mechanical closure of the os uteri. He has collected many specimens of uteri where the orifice barely admitted the probe, and where, as might be expected, the menstruation has always been painful. The cure of such cases is, of course, puncture and dilation by different sized bougies. It is important to know that such cases of painful menstruation occasionally exist, yet I am convinced that this forms a very fractional cause of a disease which is perhaps more common than any to which females are liable."

We cannot say that the therapeutic part of Mr. Wilson's paper amounts to much, but every attempt to settle the pathology of this obscure and interesting disorder is entitled to the attention of the profession.

